# Syntheses and structural characterizations of the first coordination polymers assembled from the Ni(cyclam)^2+^ cation and the benzene-1,3,5-tri­carboxyl­ate linker

**DOI:** 10.1107/S2056989022009860

**Published:** 2022-10-13

**Authors:** Sergey P. Gavrish, Sergiu Shova, Yaroslaw D. Lampeka

**Affiliations:** a L. V. Pisarzhevskii Institute of Physical Chemistry of the National Academy of Sciences of Ukraine, Prospekt Nauki 31, Kyiv, 03028, Ukraine; b "Petru Poni" Institute of Macromolecular Chemistry, Department of Inorganic Polymers, Aleea Grigore Ghika Voda 41A, RO-700487 Iasi, Romania; University of Aberdeen, Scotland

**Keywords:** crystal structure, coordination polymers, cyclam, nickel, benzene­tri­carboxyl­ate, hydrogen bonds

## Abstract

The asymmetric unit of *catena*-poly[[[(1,4,8,11-tetra­aza­cyclo­tetra­decane-κ^4^ *N* ^1^,*N* ^4^,*N* ^8^,*N* ^11^)nickel(II)]-μ_2_-5-carb­oxy­benzene-1,3-di­carboxyl­ato-κ^2^ *O* ^1^:*O* ^3^] octa­hydrate], {[Ni(C_9_H_4_O_6_)(C_10_H_24_N_4_)]·8H_2_O}_*n*_ (**I**), consists of a macrocyclic Ni^2+^ cation, a carboxyl­ate dianion and eight highly disordered water mol­ecules of crystallization. The components of the compound *catena*-poly[[[(1,4,8,11-tetra­aza­cyclo­tetra­decane-κ^4^ *N* ^1^,*N* ^4^,*N* ^8^,*N* ^11^)nickel(II)]-μ_2_-5-carb­oxy­benzene-1,3-di­carboxyl­ato-κ^2^ *O* ^1^:*O* ^3^] monohydrate], {[Ni(C_9_H_4_O_6_)(C_10_H_24_N_4_)]·H_2_O}_*n*_ (**II**), are two crystallographically unique centrosymmetric macrocyclic dications, a carboxyl­ate dianion and one water mol­ecule of crystallization. In each compound, the metal ion is coordinated in the equatorial plane by the four secondary N atoms of the macrocyclic ligand, which adopts the most energetically stable *trans*-III conformation, and two mutually *trans* O atoms of the carboxyl­ate anions in a slightly tetra­gonally distorted *trans*-NiN_4_O_2_ octa­hedral geometry. The crystals of both compounds are composed of parallel coordination polymeric chains running along the [010] direction in **I** and the [110] and [1  0] directions in **II**. The bridging carboxyl­ate anions display different modes of coordination connected with the relative orientation of coordinated O atoms, *i.e.*, remote in **I** and inter­mediate in **II**, thus resulting in essentially different distances between the Ni atoms in the chains [11.0657 (4) and 8.9089 (2) Å in **I** and **II**, respectively]. As a result of hydrogen-bonding inter­actions, the chains are joined together in sheets oriented parallel to the (10  ) and (001) planes in **I** and **II**, respectively.

## Abstract 2

The coordination polyhedra of the complex cations in the one-dimensional coordination polymers **I** and **II** represent tetra­gonally distorted NiN_4_O_2_ octa­hedra with the four N atoms of the aza­macrocyclic cyclam ligand in the equatorial planes and two O atoms of the benzene­tri­carboxyl­ate anions in the axial positions. The crystals of both compounds are composed of parallel coordination polymeric chains running along the [010] direction in **I** and the [110] and [1 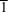 0] directions in **II**. As a result of hydrogen-bonding inter­actions, the chains are joined together in layers oriented parallel to the (10 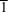 ) and (001) planes in **I** and **II**, respectively.

## 1. Chemical context

Aza­macrocyclic complexes of transition metals are widely used for the construction of metal–organic frameworks (MOFs) – crystalline porous materials displaying many promising properties connected with the possibilities of their practical applications (Lampeka & Tsymbal, 2004 ▸; Suh & Moon, 2007 ▸; Suh *et al.*, 2012 ▸; Stackhouse & Ma, 2018 ▸). Complexes of the 14-membered tetra­aza cyclam ligand (cyclam = 1,4,8,11-tetra­aza­cyclo­tetra­decane, C_10_H_24_N_4_, *L*), which is the most suitable for binding of 3*d* transition-metal ions, in particular, Ni^2+^, are among popular metal-containing nodes in the formation of MOFs. Their inter­actions with different oligo­carboxyl­ates as the most common bridging ligands (Rao *et al.*, 2004 ▸) usually result in coordination polymers, the dimensionalities of which are dependent on the number of carb­oxy­lic groups present in the linker. As was shown formerly for a number of macrocyclic Ni^2+^ complexes of aza- and di­aza­cyclam derivatives, which are the closest structural analogues of *L* (aza­cyclam = 1,3,5,8,12-penta­tetra­aza­cyclo­tetra­decane, di­aza­cyclam = 1,3,5,8,10,12-hexa­aza­zacyclo­tetra­deca­ne), the coordination of the simplest tridentate aromatic ligand benzene-1,3,5-tri­carboxyl­ate (btc^3–^) in the *trans*-axial coordination positions of the metal ion leads to the formation of two-dimensional coordination polymers with hexa­gonal nets of 6^3^ topology (Choi *et al.*, 2001 ▸; Meng *et al.*, 2011 ▸; Choi & Suh, 1998 ▸; Ryoo *et al.*, 2010 ▸; Lu *et al.*, 2001 ▸; Lu *et al.*, 2002 ▸; Lampeka *et al.*, 2012 ▸). Surprisingly, for the Ni(*L*)^2+^ cation itself, only ionic compounds built on the *trans*-di­aqua [Ni(*L*)(H_2_O)_2_]^2+^ cation and the non-coordinated btc^3−^anion have been described to date (Choi *et al.*, 1999 ▸; Parsons *et al.*, 2006 ▸; Tadokoro *et al.*, 2015 ▸). 
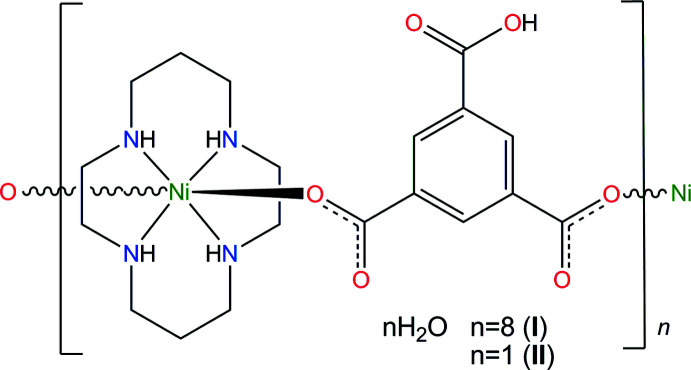


The present work describes the preparation and structural characterization of the first representatives of polymeric complexes formed by Ni(*L*)^2+^ and the benzene-1,3,5-tri­carboxyl­ate anion, namely, *catena*-poly[[[(1,4,8,11-tetra­aza­cyclo­tetra­decane-κ^4^ *N* ^1^,*N* ^4^,*N* ^8^,*N* ^11^)nickel(II)]-μ_2_-5-carb­oxy­benzene-1,3-di­carboxyl­ato-κ^2^ *O* ^1^:*O* ^3^] octa­hydrate], {[Ni(C_9_H_4_O_6_)(C_10_H_24_N_4_)]·8H_2_O}_*n*_ (**I**) and *catena*-poly[[[(1,4,8,11-tetra­aza­cyclo­tetra­decane-κ^4^ *N* ^1^,*N* ^4^,*N* ^8^,*N* ^11^)nickel(II)]-μ_2_-5-carb­oxy­benzene-1,3-di­carb­oxyl­ato-κ^2^ *O* ^1^:*O* ^3^] monohydrate], {[Ni(C_9_H_4_O_6_)(C_10_H_24_N_4_)]·H_2_O}_*n*_ (**II**).

## 2. Structural commentary

**Figure 1:**
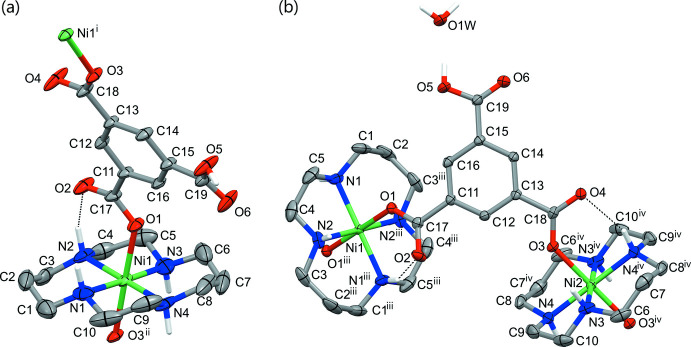
The extended asymmetric unit in (*a*) **I** and (*b*) **II** showing the coordination environment of the Ni atoms and the atom-labeling scheme (displacement ellipsoids are drawn at the 30% probability level). C-bound H atoms are omitted for clarity. Intra­molecular hydrogen bonds are shown as dotted lines. Symmetry codes: (i) −*x* + 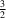 , *y* − 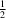 , −*z* + 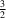 ; (ii) −*x* + 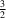 , *y* + 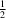 , −*z* + 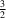 ; (iii) −*x* + 1, −*y*, −*z* + 1; (iv) −*x*, −*y* + 1, −*z* + 1.

The mol­ecular structures of **I** and **II** are shown in Fig. 1 ▸. The asymmetric unit of **I** consists of a macrocyclic [Ni(*L*)]^2+^ di-cation, a monoprotonated carboxyl­ate Hbtc^2−^ dianion and eight water mol­ecules of crystallization, while the components of **II** are the same dianion, two crystallographically unique centrosymmetric dications and one water mol­ecule of crystallization. The coordination polyhedra of the metal ions in both complexes are very similar: the Ni^2+^ ions are coordinated by the four secondary N atoms of the macrocycle *L*, which adopt the most energetically stable *trans*-III (*R,R,S,S*) conformation (Bosnich *et al.*, 1965*a*; Barefield *et al.*, 1986) in which the five-membered (N—Ni—N bite angles ≃ 85°) and six-membered (N—Ni—N bite angles ≃ 95°) chelate rings are in *gauche* and *chair* conformations, respectively (Table 1 ▸). The O atoms of the carboxyl­ate ligands occupy the axial positions in the coordination spheres of the metal ions, resulting in a tetra­gonally elongated *trans*-NiN_4_O_2_ coordination octa­hedra with the Ni—N bond lengths (average value 2.063 Å) slightly shorter than the Ni—O ones (average value 2.121 Å) (Table 1 ▸). The axial Ni—O bonds are nearly orthogonal to the NiN_4_ planes (deviations of the angles N—Ni—O from 90° do not exceed 5°). The deviations of the Ni and N atoms from the mean N_4_ plane in **I** are 0.011 Å and ±0.009 Å, respectively, while the NiN_4_ coordination moieties in **II** are strictly planar because of the location of the metal ions on crystallographic inversion centers. As in other complexes of the Ni^2+^ macrocyclic cations and carboxyl­ate ligands (Tsymbal *et al.*, 2021 ▸) the Ni—O bonds in **I** and **II** are reinforced by the intra­molecular hydrogen bonds between the secondary NH atoms and the non-coordinated O atoms of each coordinated carb­oxy­lic group (Fig. 1 ▸, Tables 2 ▸ and 3 ▸).

The C—O bond lengths in the deprotonated carboxyl­ate groups are nearly equal, thus indicating essential electron delocalization, while protonated carb­oxy­lic groups remain non-delocalized [the lengths of the C—OH and C=O bonds in **I** and **II** are 1.305 (4) and 1.200 (3) Å and 1.314 (4) and 1.205 (3) Å, respectively]. The mean planes of the carboxyl­ate groups are slightly tilted relative to the mean plane of their attached aromatic rings (average angle equals 7.0° in **I** and 16.0° in **II**).

**Figure 2:**
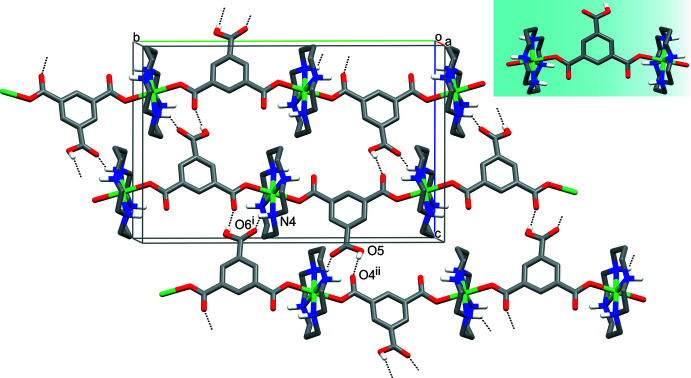
The hydrogen-bonded (dashed lines) sheet in **I**. C-bound H atoms have been omitted; the intra­molecular hydrogen bonds are not shown. The mode of coordination of carboxyl­ate ligand is shown as an inset. Symmetry codes: (i) −*x* + 2, −*y* + 1, −*z* + 2; (ii) *x* + 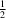 , −*y* + 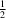 , *z* + 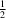 .

In both complexes, the monoprotonated carboxyl­ate ligands display a μ_2_-bis-monodentate bridging function of the isophthalate type, resulting in the formation of one-dimensional coordination polymers (Figs. 2 ▸ and 3 ▸). The Ni—O coordination bonds of the Hbtc^2−^ bridge are characterized by the *syn/syn* orientation. Since the carboxyl­ate groups are nearly coplanar with the aromatic rings, the possibility arises for appearance of different modes of ligand coordination, depending on the mutual spatial arrangement of coordinated O atoms (Tsymbal *et al.*, 2021 ▸). In the complexes under consideration, these modes can be recognized as remote (rm) in **I** and inter­mediate (im) in **II** (see insets in Figs. 2 ▸ and 3 ▸).

Such peculiarities lead to several differences in the structures of the polymeric chains. In particular, the angle between the mean NiN_4_ planes of the macrocyclic cations in **I** is 40.62 (1)°, while they are nearly orthogonal in **II** [85.49 (1)°]. Therewith, the chains of the Ni atoms in **I** are non-linear [the angle Ni⋯Ni⋯Ni is 169.590 (9)°], in contrast to strictly linear metal atom chains in **II**. The most important difference is connected with the mode of the carboxyl­ate coordination and consists of essentially different distances between the Ni atoms in the chains formed by the rm-*syn/syn* coordinated ligand in **I** [Ni⋯Ni = 11.0657 (4) Å], as compared to the im-*syn/syn* coordinated one in **II** [8.9089 (2) Å].

## 3. Supra­molecular features

Both compounds are characterized by lamellar structures as the result of linking of the polymeric chains into sheets due to hydrogen-bonding inter­actions (Tables 2 ▸ and 3 ▸). The key role in the formation of sheets oriented parallel to the (10 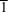 ) plane from the chains running along the [010] direction in the crystals of **I** is played by the protonated carb­oxy­lic group of the Hbtc^2−^ dianion, which forms two O—H⋯O hydrogen bonds acting both as the proton donor in a strong inter­action with the O atom of the coordinated carb­oxy­lic ligand on neighboring chain [O5—H5⋯O4(*x* + 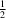 , −*y* + 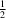 , *z* + 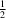 )] and as the proton acceptor in a weak inter­action with the secondary amino group of the macrocyclic cation belonging to the same neighboring chain [N4—H4⋯O6(−*x* + 2, −*y* + 1, −*z* + 2)] (Fig. 2 ▸). There are no hydrogen-bonding contacts between the sheets and the three-dimensional coherence of the crystal is provided by van der Waals inter­actions.

**Figure 3:**
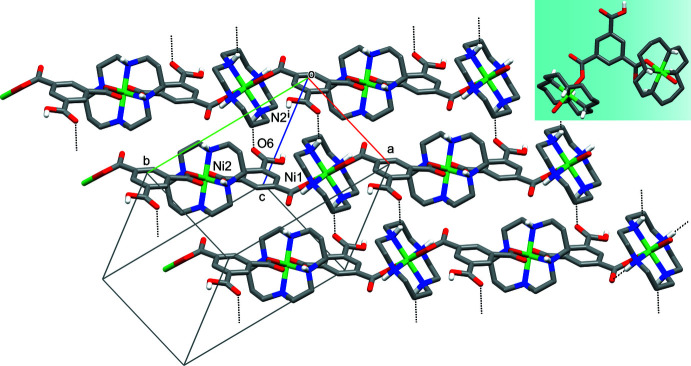
The hydrogen-bonded (dashed lines) sheet in **II**. C-bound H atoms and water mol­ecule of crystallization have been omitted; the intra­molecular hydrogen bonds are not shown. The mode of coordination of carboxyl­ate ligand is shown as an inset. Symmetry code: (i) *x* − 1, *y*, *z*.

In the crystal of **II**, polymeric chains with different orientations are present, namely, running along the [110] or [1 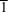 0] directions. As a result of the weak hydrogen bond between the carbonyl O6 atom of the protonated carb­oxy­lic group of the acid as the acceptor and the secondary N2—H2 amino group of the macrocyclic cation of a neighboring chain as the donor (Fig. 3 ▸), they form alternating sheets oriented parallel to the (001) plane. At the same time, the hydroxyl group of the protonated carboxyl­ate group as the donor inter­acts strongly with the water mol­ecule of crystallization as acceptor, and this inter­action together with two additional hydrogen bonds with participation of O1*W* mol­ecule results in a three-dimensional network in **II**.

As estimated by *PLATON* (Spek, 2020 ▸), the volume of the solvent-accessible voids in **I** in the form of parallel one-dimensional channels equals 1111 Å^3^ (37.5% of the unit-cell volume) and according to SQUEEZE calculations it is filled with eight highly disordered water mol­ecules of crystallization. The crystals of **II** are non-porous.

## 4. Database survey

The Cambridge Structural Database (CSD, Version 5.43, last update March 2022; Groom *et al.*, 2016 ▸) contains descriptions of several polymorphs of compounds containing the Ni(*L*) moiety and the benzene-1,3,5-tri­carboxyl­ate anion (refcodes GOQTIP, Choi *et al.*, 1999 ▸; PELCOZ, Parsons *et al.*, 2006 ▸; GOQTIP01, SABLEP, SABLOZ and SABLOZ01, Tadokoro *et al.*, 2015 ▸). All of them are highly hydrated (18–29 water mol­ecules of crystallization) ionic complexes containing the *trans*-di­aqua [Ni(*L*)(H_2_O)_2_]^2+^ dication and non-coordinated btc^3–^ trianions. At the same time, a number of two-dimensional coordination polymers built on parent 14-membered derivatives of Ni(aza­cyclam) (CAXMIZ, Lampeka *et al.*, 2012 ▸) and Ni(di­aza­cyclam) (IPOZIW, Choi *et al.*, 2001 ▸; IWESIN and IWESOT, Meng *et al.*, 2011 ▸; JEDQIS and JEDQOY, Choi & Suh, 1998 ▸; UJUHUD, Ryoo *et al.*, 2010 ▸; VOQSAV, Lu *et al.*, 2001 ▸; and WUJDEK, Lu *et al.*, 2002 ▸) bearing different substituents at the non-coordinated distal nitro­gen atom(s) have been structurally characterized. In addition, two compounds with other structures have been described. One represents the mol­ecular complex in which the *trans*-[Ni(*L^A^*)(btc)_2_]^4–^ anion compensates the charge of the two *trans*-[Ni(*L^A^*)(H_2_O)_2_]^2+^ cations (*L^A^* = 3,10-dibutyl-1,3,5,8,10,12-hexa­aza­cyclo­tetra­deca­ne) (SUXXEQ, Shin *et al.*, 2016 ▸), and the other is the hydrated (3.5 water mol­ecules of crystallization) one-dimensional coordination polymer formed by the [Ni(*L^B^*)]^2+^ cation and the μ_2_ Hbtc^2–^ linker (*L^B^* = 1,3,6,9,11,14-hexa­aza­tri­cyclo­[12.2.1.1^6,9^]octa­deca­ne) (SEF­LOG, Tao *et al.*, 2012 ▸). The structure of the latter is similar to the structure of **I** – it is a neutral one-dimensional coordination polymer with parallel alignment of the chains formed due to the carboxyl­ate displaying the rm-*syn/syn* mode of the bridging function. Correspondingly, the Ni⋯Ni distance in this compound (11.313 Å) is close to that observed in **I**, though the chains, in contrast to **I**, are linear.

## 5. Synthesis and crystallization

All chemicals and solvents used in this work were purchased from Sigma–Aldrich and used without further purification. The complex [Ni(*L*)](ClO_4_)_2_ was prepared from ethanol solution as described in the literature (Bosnich *et al.*, 1965 ▸).

The complex **[Ni(** ***L*** **)(Hbtc)·8H_2_O]**, (**I**), was prepared as follows. [Ni(*L*)](ClO_4_)_2_ (153 mg, 0.33 mmol) and H_3_btc (50 mg, 0.24 mmol) were dissolved in 10 ml of a DMF/H_2_O mixture (4:1 by volume) and the solution was heated at 358 K for 30 h. A small amount of pink needle-like crystals in the form of concretions was formed in a week. These were filtered off, washed with small amounts of methanol and diethyl ether, and dried in air. Yield: 15 mg (10% based on acid). Analysis calculated for C_19_H_44_N_4_NiO_14_: C 37.36, H 7.27, N 9.18%. Found: C 37.52, H 7.31, N 9.15%. Single crystals of **I** suitable for X-ray diffraction analysis were selected from the sample formed after refrigerating the mother liquor for several days.

Apparently, the complex **[Ni(** ***L*** **)(Hbtc)·H_2_O]**, (**II**), is more thermodynamically stable than **I** and it was prepared according to similar procedure, except that initially precipitated crystals were left to remain under the mother liquor at ambient temperature. Over *ca* one week, the needle-like crystals of **I** dissolved; instead, a precipitate in the form of rhomb-shaped plates was formed and single crystals of **II** suitable for X-ray diffraction analysis were selected from this reaction mixture. Alternatively, larger amounts of **II** can be obtained using an analogous procedure but using higher concentrations of the reagents. [Ni(*L*)](ClO_4_)_2_ (200 mg, 0.44 mmol) and H_3_btc (65 mg, 0.31 mmol) were dissolved in 10 ml of a DMF/H_2_O mixture (4:1 by volume) and the solution was heated at 358 K for 24 h. After cooling of the reaction mixture, the product began to crystallize in several hours in the form of pink plate-like concretions. It was filtered off, washed with small amounts of methanol and diethyl ether, and dried in air. Yield: 38 mg (25% based on acid). Analysis calculated for C_19_H_30_N_4_NiO_7_: C 47.09, H 6.24, N 11.57%. Found: C 47.15, H 6.31, N 11.65%.

**Caution! Perchlorate salts of metal complexes are potentially explosive and should be handled with care.**

## 6. Refinement

Crystal data, data collection and structure refinement details are summarized in Table 4 ▸. The H atoms in **I** and **II** were placed in geometrically idealized positions and constrained to ride on their parent atoms, with C—H distances of 0.93 Å (ring H atoms), 0.97 Å (methyl­ene H atoms), N—H distances of 0.98 Å, O—H distances of 0.82 Å (protonated carboxyl­ate group) and 0.85 Å (water mol­ecules) with *U*_iso_(H) values of 1.2*U*_eq_ or 1.5*U*_eq_ times those of the corresponding parent atoms. SQUEEZE calculations indicate the presence of eight water mol­ecules of crystallization per asymmetric unit of **I**.

## Supplementary Material

Crystal structure: contains datablock(s) I, II. DOI: 10.1107/S2056989022009860/hb8040sup1.cif

Structure factors: contains datablock(s) I. DOI: 10.1107/S2056989022009860/hb8040Isup2.hkl

Structure factors: contains datablock(s) II. DOI: 10.1107/S2056989022009860/hb8040IIsup3.hkl

CCDC references: 2210108, 2210107

Additional supporting information:  crystallographic information; 3D view; checkCIF report

## Figures and Tables

**Table 1:** Selected geometric parameters (Å, °)

**I**		**II**	
Ni1—N1	2.056 (3)	Ni1—N1	2.051 (2)
Ni1—N2	2.066 (2)	Ni1—N2	2.064 (3)
Ni1—N3	2.053 (3)	Ni2—N3	2.063 (2)
Ni1—N4	2.046 (3)	Ni2—N4	2.050 (3)
Ni1—O1	2.1106 (18)	Ni1—O1	2.1242 (19)
Ni1—O3i	2.1377 (18)	Ni2—O3	2.1129 (18)
			
N1—Ni1—N4	85.55 (15)	N1—Ni1—N2	85.31 (11)
N2—Ni1—N3	84.92 (14)	N3—Ni2—N4	85.38 (11)
N1—Ni1—N2	93.13 (14)	N1—Ni1—N2ii	94.69 (11)
N3—Ni1—N4	96.41 (15)	N3—Ni1—N4iii	94.62 (11)

Symmetry codes: (i) −*x* + 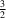 , *y* + 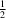 , −*z* + 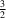 ; (ii) −*x* + 1, −*y*, −*z* + 1; (iii) −*x*, −*y* + 1, −*z* + 1.

**Table 2:** Hydrogen-bond geometry (Å, °) for **I** [Chem scheme1]

*D*—H⋯*A*	*D*—H	H⋯*A*	*D*⋯*A*	*D*—H⋯*A*
O5—H5⋯O4^i^	0.82	1.83	2.604 (3)	157
N2—H2⋯O2	0.98	2.08	2.969 (3)	150
N4—H4⋯O4^ii^	0.98	2.00	2.891 (3)	151
N4—H4⋯O6^iii^	0.98	2.59	3.226 (4)	123

Symmetry codes: (i) 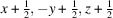 ; (ii) 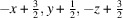 ; (iii) 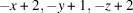 .

**Table 3:** Hydrogen-bond geometry (Å, °) for **II** [Chem scheme1]

*D*—H⋯*A*	*D*—H	H⋯*A*	*D*⋯*A*	*D*—H⋯*A*
O5—H5⋯O1*W*	0.82	1.72	2.531 (3)	170
N2—H2⋯O6^i^	0.98	2.38	3.199 (4)	141
N4—H4⋯O4^ii^	0.98	2.09	2.959 (3)	147
N1—H1⋯O2^iii^	0.98	1.97	2.872 (3)	153
O1*W*—H1*WA*⋯O2^iv^	0.85	1.83	2.664 (3)	168
O1*W*—H1*WB*⋯O4^v^	0.85	1.92	2.747 (3)	165

Symmetry codes: (i) 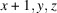 ; (ii) 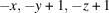 ; (iii) 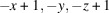 ; (iv) 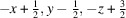 ; (v) 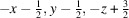 .

**Table 4:** Experimental details

	**I**	**II**
Crystal data		
Chemical formula	[Ni(C_9_H_4_O_6_)(C_10_H_24_N_4_)]·8H_2_O	[Ni(C_9_H_4_O_6_)(C_10_H_24_N_4_)]·H_2_O
*M*_r_	467.16	485.18
Crystal system, space group	Monoclinic, *P*2_1_/*n*	Monoclinic, *P*2_1_/*n*
Temperature (K)	293	293
*a*, *b*, *c* (Å)	9.3650 (6), 22.0401 (8), 14.3567 (7)	9.3852 (3), 15.1459 (4), 15.7561 (5)
β (°)	91.457 (5)	98.604 (3)
*V* (Å^3^)	2962.3 (3)	2214.49 (12)
*Z*	4	4
Radiation type	Mo *K*α	Mo *K*α
μ (mm^−1^)	0.69	0.92
Crystal size (mm)	0.40 × 0.20 × 0.10	0.20 × 0.20 × 0.07

Data collection		
Diffractometer	Rigaku Xcalibur Eos	Rigaku Xcalibur Eos
Absorption correction	Multi-scan (*CrysAlis PRO*; Rigaku OD, 2021 ▸)	Multi-scan (*CrysAlis PRO*; Rigaku OD, 2021 ▸)
*T*_min_, *T*_max_	0.751, 1.000	0.988, 1.000
No. of measured, independent and observed [*I* > 2σ(*I*)] reflections	21516, 6875, 3858	14088, 4534, 2876
*R*_int_	0.058	0.042
(sin θ/λ)_max_ (Å^−1^)	0.688	0.625

Refinement		
*R*[*F* ^2^ > 2σ(*F* ^2^)], *wR*(*F* ^2^), *S*	0.059, 0.125, 1.08	0.045, 0.108, 1.03
No. of reflections	6875	4534
No. of parameters	272	287
H-atom treatment	H-atom parameters constrained	H-atom parameters constrained
Δρ_max_, Δρ_min_ (e Å^−3^)	0.31, −0.32	0.32, −0.43

Computer programs: *CrysAlis PRO* (Rigaku OD, 2021 ▸), *SHELXS* (Sheldrick, 2015*a* ▸), *SHELXL2018/3* (Sheldrick, 2015*b* ▸), *Mercury* (Macrae *et al.*, 2020 ▸) and *publCIF* (Westrip, 2010 ▸).

## supplementary crystallographic information

### catena-Poly[[[(1,4,8,11-tetraazacyclotetradecane-κ4N1,N4,N8,N11)nickel(II)]-µ2-5-carboxybenzene-1,3-dicarboxylato-κ2O1:O3] octahydrate] (I) Crystal data

**Table:**

[Ni(C_9_H_4_O_6_)(C_10_H_24_N_4_)]·8H_2_O	*F*(000) = 984
*M**_r_* = 467.16	*D*_x_ = 1.047 Mg m^−^^3^
Monoclinic, *P*2_1_/*n*	Mo *K*α radiation, λ = 0.71073 Å
*a* = 9.3650 (6) Å	Cell parameters from 3816 reflections
*b* = 22.0401 (8) Å	θ = 1.9–23.7°
*c* = 14.3567 (7) Å	µ = 0.69 mm^−^^1^
β = 91.457 (5)°	*T* = 293 K
*V* = 2962.3 (3) Å^3^	Prism, clear light pink
*Z* = 4	0.40 × 0.20 × 0.10 mm

### catena-Poly[[[(1,4,8,11-tetraazacyclotetradecane-κ4N1,N4,N8,N11)nickel(II)]-µ2-5-carboxybenzene-1,3-dicarboxylato-κ2O1:O3] octahydrate] (I) Data collection

**Table:**

Rigaku Xcalibur Eos diffractometer	6875 independent reflections
Radiation source: fine-focus sealed X-ray tube, Enhance (Mo) X-ray Source	3858 reflections with *I* > 2σ(*I*)
Graphite monochromator	*R*_int_ = 0.058
Detector resolution: 16.1593 pixels mm^-1^	θ_max_ = 29.3°, θ_min_ = 2.6°
ω scans	*h* = −11→11
Absorption correction: multi-scan (CrysAlis PRO; Rigaku OD, 2021)	*k* = −27→27
*T*_min_ = 0.751, *T*_max_ = 1.000	*l* = −18→17
21516 measured reflections	

### catena-Poly[[[(1,4,8,11-tetraazacyclotetradecane-κ4N1,N4,N8,N11)nickel(II)]-µ2-5-carboxybenzene-1,3-dicarboxylato-κ2O1:O3] octahydrate] (I) Refinement

**Table:**

Refinement on *F*^2^	Primary atom site location: dual
Least-squares matrix: full	Hydrogen site location: inferred from neighbouring sites
*R*[*F*^2^ > 2σ(*F*^2^)] = 0.059	H-atom parameters constrained
*wR*(*F*^2^) = 0.125	*w* = 1/[σ^2^(*F*_o_^2^) + (0.0348*P*)^2^] where *P* = (*F*_o_^2^ + 2*F*_c_^2^)/3
*S* = 1.08	(Δ/σ)_max_ = 0.001
6875 reflections	Δρ_max_ = 0.31 e Å^−^^3^
272 parameters	Δρ_min_ = −0.32 e Å^−^^3^
0 restraints	

### catena-Poly[[[(1,4,8,11-tetraazacyclotetradecane-κ4N1,N4,N8,N11)nickel(II)]-µ2-5-carboxybenzene-1,3-dicarboxylato-κ2O1:O3] octahydrate] (I) Special details

**Table:**

Geometry. All esds (except the esd in the dihedral angle between two l.s. planes) are estimated using the full covariance matrix. The cell esds are taken into account individually in the estimation of esds in distances, angles and torsion angles; correlations between esds in cell parameters are only used when they are defined by crystal symmetry. An approximate (isotropic) treatment of cell esds is used for estimating esds involving l.s. planes.

### catena-Poly[[[(1,4,8,11-tetraazacyclotetradecane-κ4N1,N4,N8,N11)nickel(II)]-µ2-5-carboxybenzene-1,3-dicarboxylato-κ2O1:O3] octahydrate] (I) Fractional atomic coordinates and isotropic or equivalent isotropic displacement parameters (Å^2^)

**Table:**

	*x*	*y*	*z*	*U*_iso_*/*U*_eq_	
Ni1	0.69720 (4)	0.55885 (2)	0.75502 (3)	0.03961 (15)	
O1	0.6534 (2)	0.46933 (8)	0.79800 (14)	0.0538 (6)	
O2	0.5619 (3)	0.41875 (8)	0.67715 (16)	0.0719 (8)	
O3	0.7624 (2)	0.15022 (8)	0.78673 (14)	0.0485 (6)	
O4	0.6373 (3)	0.19208 (9)	0.67255 (18)	0.1011 (11)	
O5	0.9540 (3)	0.27371 (10)	1.04356 (19)	0.0971 (11)	
H5	1.026567	0.284652	1.072019	0.146*	
O6	0.8741 (3)	0.36664 (10)	1.06441 (17)	0.0918 (10)	
N1	0.8659 (3)	0.52742 (12)	0.6799 (3)	0.0696 (9)	
H1	0.855311	0.483290	0.675135	0.084*	
N2	0.5551 (3)	0.55160 (11)	0.6431 (2)	0.0596 (9)	
H2	0.528340	0.508716	0.638191	0.072*	
N3	0.5242 (4)	0.58978 (12)	0.8261 (2)	0.0730 (10)	
H3	0.528845	0.634199	0.825184	0.088*	
N4	0.8406 (4)	0.56357 (11)	0.8647 (2)	0.0710 (10)	
H4	0.876485	0.605323	0.866867	0.085*	
C1	0.8744 (6)	0.55061 (19)	0.5861 (4)	0.1075 (18)	
H1A	0.957482	0.533515	0.556787	0.129*	
H1B	0.886094	0.594320	0.588217	0.129*	
C2	0.7421 (7)	0.5352 (2)	0.5283 (3)	0.114 (2)	
H2A	0.762054	0.542420	0.463294	0.137*	
H2B	0.722629	0.492262	0.535306	0.137*	
C3	0.6120 (6)	0.56943 (17)	0.5518 (3)	0.0911 (17)	
H3A	0.539192	0.562589	0.503691	0.109*	
H3B	0.633845	0.612446	0.552779	0.109*	
C4	0.4290 (5)	0.58417 (17)	0.6676 (4)	0.0880 (15)	
H4A	0.348784	0.571120	0.628441	0.106*	
H4B	0.443004	0.627298	0.657987	0.106*	
C5	0.3984 (5)	0.57217 (18)	0.7675 (4)	0.0958 (16)	
H5A	0.315607	0.595338	0.785710	0.115*	
H5B	0.377847	0.529444	0.776235	0.115*	
C6	0.5197 (6)	0.5718 (2)	0.9226 (4)	0.1113 (19)	
H6A	0.502707	0.528444	0.925611	0.134*	
H6B	0.439987	0.592000	0.951231	0.134*	
C7	0.6542 (8)	0.5863 (2)	0.9777 (3)	0.129 (2)	
H7A	0.677056	0.628688	0.967197	0.154*	
H7B	0.634114	0.581925	1.043262	0.154*	
C8	0.7827 (7)	0.55005 (17)	0.9583 (3)	0.111 (2)	
H8A	0.758997	0.507277	0.961644	0.133*	
H8B	0.855740	0.558380	1.005650	0.133*	
C9	0.9589 (5)	0.52540 (18)	0.8405 (4)	0.107 (2)	
H9A	0.933373	0.482969	0.846677	0.129*	
H9B	1.040926	0.533639	0.881155	0.129*	
C10	0.9923 (5)	0.53932 (19)	0.7435 (5)	0.110 (2)	
H10A	1.072151	0.514566	0.724517	0.132*	
H10B	1.020103	0.581576	0.738609	0.132*	
C11	0.6747 (3)	0.36318 (11)	0.8010 (2)	0.0399 (8)	
C12	0.6602 (3)	0.30772 (11)	0.7576 (2)	0.0420 (8)	
H12	0.612201	0.305534	0.700194	0.050*	
C13	0.7152 (3)	0.25504 (11)	0.7973 (2)	0.0401 (8)	
C14	0.7864 (3)	0.25897 (12)	0.8827 (2)	0.0486 (9)	
H14	0.825804	0.224222	0.909616	0.058*	
C15	0.7995 (3)	0.31362 (12)	0.9283 (2)	0.0463 (8)	
C16	0.7405 (3)	0.36579 (12)	0.8868 (2)	0.0424 (8)	
H16	0.746168	0.402657	0.918165	0.051*	
C17	0.6238 (4)	0.42117 (12)	0.7530 (2)	0.0450 (8)	
C18	0.7045 (4)	0.19457 (12)	0.7481 (2)	0.0506 (9)	
C19	0.8792 (4)	0.32119 (14)	1.0186 (2)	0.0618 (11)	

### catena-Poly[[[(1,4,8,11-tetraazacyclotetradecane-κ4N1,N4,N8,N11)nickel(II)]-µ2-5-carboxybenzene-1,3-dicarboxylato-κ2O1:O3] octahydrate] (I) Atomic displacement parameters (Å^2^)

**Table:**

	*U*^11^	*U*^22^	*U*^33^	*U*^12^	*U*^13^	*U*^23^
Ni1	0.0576 (3)	0.0201 (2)	0.0402 (3)	0.00044 (18)	−0.0169 (2)	−0.00049 (15)
O1	0.0879 (18)	0.0212 (11)	0.0512 (14)	−0.0037 (11)	−0.0180 (12)	0.0000 (9)
O2	0.116 (2)	0.0268 (12)	0.0699 (18)	0.0005 (12)	−0.0539 (16)	0.0005 (10)
O3	0.0769 (16)	0.0214 (11)	0.0455 (13)	0.0068 (10)	−0.0287 (12)	−0.0031 (9)
O4	0.176 (3)	0.0313 (13)	0.091 (2)	0.0219 (15)	−0.100 (2)	−0.0163 (11)
O5	0.156 (3)	0.0365 (14)	0.094 (2)	0.0040 (15)	−0.091 (2)	−0.0084 (13)
O6	0.150 (3)	0.0500 (15)	0.0726 (19)	0.0118 (15)	−0.0564 (18)	−0.0265 (13)
N1	0.069 (2)	0.0365 (17)	0.103 (3)	−0.0020 (15)	0.012 (2)	−0.0088 (16)
N2	0.083 (2)	0.0269 (15)	0.067 (2)	−0.0068 (14)	−0.0393 (18)	0.0016 (13)
N3	0.097 (3)	0.0377 (17)	0.085 (3)	0.0057 (16)	0.018 (2)	−0.0059 (16)
N4	0.112 (3)	0.0204 (14)	0.077 (2)	−0.0101 (15)	−0.059 (2)	0.0079 (13)
C1	0.156 (5)	0.066 (3)	0.103 (4)	−0.017 (3)	0.057 (4)	−0.010 (3)
C2	0.227 (7)	0.065 (3)	0.052 (3)	−0.029 (4)	0.018 (4)	−0.009 (2)
C3	0.163 (5)	0.056 (3)	0.052 (3)	−0.026 (3)	−0.052 (3)	0.004 (2)
C4	0.079 (3)	0.056 (3)	0.127 (4)	−0.002 (2)	−0.051 (3)	0.008 (3)
C5	0.066 (3)	0.061 (3)	0.160 (5)	0.003 (2)	−0.002 (4)	−0.011 (3)
C6	0.180 (6)	0.077 (3)	0.080 (4)	−0.008 (3)	0.056 (4)	−0.007 (3)
C7	0.284 (8)	0.060 (3)	0.041 (3)	−0.014 (4)	0.004 (4)	−0.007 (2)
C8	0.232 (7)	0.041 (2)	0.055 (3)	−0.019 (3)	−0.062 (4)	0.012 (2)
C9	0.095 (4)	0.050 (3)	0.172 (6)	−0.007 (2)	−0.086 (4)	0.015 (3)
C10	0.055 (3)	0.049 (3)	0.223 (7)	−0.003 (2)	−0.022 (4)	−0.012 (3)
C11	0.055 (2)	0.0183 (16)	0.046 (2)	−0.0037 (13)	−0.0130 (16)	0.0015 (12)
C12	0.057 (2)	0.0269 (17)	0.041 (2)	0.0024 (13)	−0.0192 (16)	−0.0036 (12)
C13	0.057 (2)	0.0214 (16)	0.0409 (19)	−0.0007 (13)	−0.0190 (16)	−0.0010 (12)
C14	0.069 (2)	0.0227 (16)	0.053 (2)	−0.0013 (14)	−0.0261 (18)	0.0011 (13)
C15	0.063 (2)	0.0270 (17)	0.048 (2)	−0.0046 (14)	−0.0208 (17)	0.0009 (13)
C16	0.062 (2)	0.0214 (16)	0.043 (2)	−0.0050 (14)	−0.0119 (17)	−0.0053 (12)
C17	0.057 (2)	0.0216 (16)	0.055 (2)	−0.0046 (14)	−0.0146 (18)	0.0023 (14)
C18	0.080 (3)	0.0225 (17)	0.047 (2)	0.0004 (16)	−0.0342 (19)	−0.0003 (13)
C19	0.100 (3)	0.034 (2)	0.049 (2)	−0.0126 (19)	−0.035 (2)	0.0023 (15)

### catena-Poly[[[(1,4,8,11-tetraazacyclotetradecane-κ4N1,N4,N8,N11)nickel(II)]-µ2-5-carboxybenzene-1,3-dicarboxylato-κ2O1:O3] octahydrate] (I) Geometric parameters (Å, º)

**Table:**

Ni1—O1	2.1106 (18)	C3—H3B	0.9700
Ni1—O3^i^	2.1377 (18)	C4—H4A	0.9700
Ni1—N1	2.056 (3)	C4—H4B	0.9700
Ni1—N2	2.066 (2)	C4—C5	1.494 (6)
Ni1—N3	2.053 (3)	C5—H5A	0.9700
Ni1—N4	2.046 (3)	C5—H5B	0.9700
O1—C17	1.270 (3)	C6—H6A	0.9700
O2—C17	1.221 (3)	C6—H6B	0.9700
O3—C18	1.241 (3)	C6—C7	1.505 (7)
O4—C18	1.241 (3)	C7—H7A	0.9700
O5—H5	0.8200	C7—H7B	0.9700
O5—C19	1.305 (4)	C7—C8	1.477 (7)
O6—C19	1.200 (3)	C8—H8A	0.9700
N1—H1	0.9800	C8—H8B	0.9700
N1—C1	1.445 (5)	C9—H9A	0.9700
N1—C10	1.499 (5)	C9—H9B	0.9700
N2—H2	0.9800	C9—C10	1.467 (6)
N2—C3	1.481 (5)	C10—H10A	0.9700
N2—C4	1.434 (5)	C10—H10B	0.9700
N3—H3	0.9800	C11—C12	1.377 (3)
N3—C5	1.481 (5)	C11—C16	1.364 (4)
N3—C6	1.443 (5)	C11—C17	1.523 (4)
N4—H4	0.9800	C12—H12	0.9300
N4—C8	1.492 (5)	C12—C13	1.387 (3)
N4—C9	1.441 (6)	C13—C14	1.383 (4)
C1—H1A	0.9700	C13—C18	1.511 (4)
C1—H1B	0.9700	C14—H14	0.9300
C1—C2	1.512 (7)	C14—C15	1.375 (4)
C2—H2A	0.9700	C15—C16	1.403 (4)
C2—H2B	0.9700	C15—C19	1.488 (4)
C2—C3	1.479 (6)	C16—H16	0.9300
C3—H3A	0.9700		
			
O1—Ni1—O3^i^	178.71 (9)	C5—C4—H4B	109.9
N1—Ni1—O1	89.78 (10)	N3—C5—C4	109.3 (4)
N1—Ni1—O3^i^	91.51 (10)	N3—C5—H5A	109.8
N1—Ni1—N2	93.13 (14)	N3—C5—H5B	109.8
N2—Ni1—O1	91.66 (9)	C4—C5—H5A	109.8
N2—Ni1—O3^i^	88.28 (8)	C4—C5—H5B	109.8
N3—Ni1—O1	90.19 (10)	H5A—C5—H5B	108.3
N3—Ni1—O3^i^	88.52 (10)	N3—C6—H6A	108.8
N3—Ni1—N1	178.04 (14)	N3—C6—H6B	108.8
N3—Ni1—N2	84.92 (14)	N3—C6—C7	113.7 (4)
N4—Ni1—O1	87.21 (9)	H6A—C6—H6B	107.7
N4—Ni1—O3^i^	92.89 (8)	C7—C6—H6A	108.8
N4—Ni1—N1	85.55 (15)	C7—C6—H6B	108.8
N4—Ni1—N2	178.25 (12)	C6—C7—H7A	107.9
N4—Ni1—N3	96.41 (15)	C6—C7—H7B	107.9
C17—O1—Ni1	132.4 (2)	H7A—C7—H7B	107.2
C18—O3—Ni1^ii^	134.01 (19)	C8—C7—C6	117.4 (4)
C19—O5—H5	109.5	C8—C7—H7A	107.9
Ni1—N1—H1	107.1	C8—C7—H7B	107.9
C1—N1—Ni1	115.5 (3)	N4—C8—H8A	109.2
C1—N1—H1	107.1	N4—C8—H8B	109.2
C1—N1—C10	116.4 (4)	C7—C8—N4	112.2 (3)
C10—N1—Ni1	103.2 (3)	C7—C8—H8A	109.2
C10—N1—H1	107.1	C7—C8—H8B	109.2
Ni1—N2—H2	106.8	H8A—C8—H8B	107.9
C3—N2—Ni1	115.4 (2)	N4—C9—H9A	110.3
C3—N2—H2	106.8	N4—C9—H9B	110.3
C4—N2—Ni1	106.8 (2)	N4—C9—C10	106.9 (4)
C4—N2—H2	106.8	H9A—C9—H9B	108.6
C4—N2—C3	113.7 (3)	C10—C9—H9A	110.3
Ni1—N3—H3	106.8	C10—C9—H9B	110.3
C5—N3—Ni1	104.9 (3)	N1—C10—H10A	109.5
C5—N3—H3	106.8	N1—C10—H10B	109.5
C6—N3—Ni1	115.4 (3)	C9—C10—N1	110.9 (4)
C6—N3—H3	106.8	C9—C10—H10A	109.5
C6—N3—C5	115.5 (4)	C9—C10—H10B	109.5
Ni1—N4—H4	106.9	H10A—C10—H10B	108.0
C8—N4—Ni1	115.9 (3)	C12—C11—C17	120.9 (3)
C8—N4—H4	106.9	C16—C11—C12	118.9 (2)
C9—N4—Ni1	106.2 (2)	C16—C11—C17	120.2 (2)
C9—N4—H4	106.9	C11—C12—H12	119.1
C9—N4—C8	113.6 (3)	C11—C12—C13	121.7 (3)
N1—C1—H1A	109.3	C13—C12—H12	119.1
N1—C1—H1B	109.3	C12—C13—C18	121.8 (2)
N1—C1—C2	111.6 (4)	C14—C13—C12	118.5 (2)
H1A—C1—H1B	108.0	C14—C13—C18	119.6 (2)
C2—C1—H1A	109.3	C13—C14—H14	119.6
C2—C1—H1B	109.3	C15—C14—C13	120.8 (3)
C1—C2—H2A	108.4	C15—C14—H14	119.6
C1—C2—H2B	108.4	C14—C15—C16	119.1 (3)
H2A—C2—H2B	107.5	C14—C15—C19	123.4 (3)
C3—C2—C1	115.4 (4)	C16—C15—C19	117.5 (2)
C3—C2—H2A	108.4	C11—C16—C15	120.9 (2)
C3—C2—H2B	108.4	C11—C16—H16	119.5
N2—C3—H3A	109.1	C15—C16—H16	119.5
N2—C3—H3B	109.1	O1—C17—C11	114.2 (3)
C2—C3—N2	112.5 (3)	O2—C17—O1	125.6 (3)
C2—C3—H3A	109.1	O2—C17—C11	120.2 (2)
C2—C3—H3B	109.1	O3—C18—C13	117.6 (2)
H3A—C3—H3B	107.8	O4—C18—O3	124.2 (3)
N2—C4—H4A	109.9	O4—C18—C13	118.3 (2)
N2—C4—H4B	109.9	O5—C19—C15	113.8 (3)
N2—C4—C5	109.0 (3)	O6—C19—O5	123.2 (3)
H4A—C4—H4B	108.3	O6—C19—C15	123.0 (3)
C5—C4—H4A	109.9		
			
Ni1—O1—C17—O2	−29.8 (5)	C10—N1—C1—C2	179.3 (4)
Ni1—O1—C17—C11	149.4 (2)	C11—C12—C13—C14	−0.1 (5)
Ni1^ii^—O3—C18—O4	9.4 (6)	C11—C12—C13—C18	−177.5 (3)
Ni1^ii^—O3—C18—C13	−171.6 (2)	C12—C11—C16—C15	3.5 (5)
Ni1—N1—C1—C2	−59.5 (4)	C12—C11—C17—O1	−175.0 (3)
Ni1—N1—C10—C9	40.3 (4)	C12—C11—C17—O2	4.1 (5)
Ni1—N2—C3—C2	56.1 (4)	C12—C13—C14—C15	1.5 (5)
Ni1—N2—C4—C5	−40.1 (4)	C12—C13—C18—O3	176.8 (3)
Ni1—N3—C5—C4	−42.2 (4)	C12—C13—C18—O4	−4.2 (5)
Ni1—N3—C6—C7	52.7 (5)	C13—C14—C15—C16	−0.4 (5)
Ni1—N4—C8—C7	−51.9 (4)	C13—C14—C15—C19	−177.7 (3)
Ni1—N4—C9—C10	45.1 (4)	C14—C13—C18—O3	−0.7 (5)
N1—C1—C2—C3	72.3 (5)	C14—C13—C18—O4	178.4 (3)
N2—C4—C5—N3	57.0 (4)	C14—C15—C16—C11	−2.1 (5)
N3—C6—C7—C8	−71.3 (6)	C14—C15—C19—O5	10.1 (5)
N4—C9—C10—N1	−59.4 (4)	C14—C15—C19—O6	−169.3 (4)
C1—N1—C10—C9	167.9 (4)	C16—C11—C12—C13	−2.4 (5)
C1—C2—C3—N2	−70.2 (5)	C16—C11—C17—O1	2.6 (5)
C3—N2—C4—C5	−168.5 (3)	C16—C11—C17—O2	−178.2 (3)
C4—N2—C3—C2	179.9 (3)	C16—C15—C19—O5	−167.3 (3)
C5—N3—C6—C7	175.5 (4)	C16—C15—C19—O6	13.4 (6)
C6—N3—C5—C4	−170.4 (3)	C17—C11—C12—C13	175.2 (3)
C6—C7—C8—N4	69.6 (6)	C17—C11—C16—C15	−174.2 (3)
C8—N4—C9—C10	173.6 (3)	C18—C13—C14—C15	179.0 (3)
C9—N4—C8—C7	−175.2 (4)	C19—C15—C16—C11	175.3 (3)

Symmetry codes: (i) −*x*+3/2, *y*+1/2, −*z*+3/2; (ii) −*x*+3/2, *y*−1/2, −*z*+3/2.

### catena-Poly[[[(1,4,8,11-tetraazacyclotetradecane-κ4N1,N4,N8,N11)nickel(II)]-µ2-5-carboxybenzene-1,3-dicarboxylato-κ2O1:O3] octahydrate] (I) Hydrogen-bond geometry (Å, º)

**Table:**

*D*—H···*A*	*D*—H	H···*A*	*D*···*A*	*D*—H···*A*
O5—H5···O4^iii^	0.82	1.83	2.604 (3)	157
N2—H2···O2	0.98	2.08	2.969 (3)	150
N4—H4···O4^i^	0.98	2.00	2.891 (3)	151
N4—H4···O6^iv^	0.98	2.59	3.226 (4)	123

Symmetry codes: (i) −*x*+3/2, *y*+1/2, −*z*+3/2; (iii) *x*+1/2, −*y*+1/2, *z*+1/2; (iv) −*x*+2, −*y*+1, −*z*+2.

### catena-Poly[[[(1,4,8,11-tetraazacyclotetradecane-κ4N1,N4,N8,N11)nickel(II)]-µ2-5-carboxybenzene-1,3-dicarboxylato-κ2O1:O3] monohydrate] (II) Crystal data

**Table:**

[Ni(C_9_H_4_O_6_)(C_10_H_24_N_4_)]·H_2_O	*F*(000) = 1024
*M**_r_* = 485.18	*D*_x_ = 1.455 Mg m^−^^3^
Monoclinic, *P*2_1_/*n*	Mo *K*α radiation, λ = 0.71073 Å
*a* = 9.3852 (3) Å	Cell parameters from 3646 reflections
*b* = 15.1459 (4) Å	θ = 2.4–25.9°
*c* = 15.7561 (5) Å	µ = 0.92 mm^−^^1^
β = 98.604 (3)°	*T* = 293 K
*V* = 2214.49 (12) Å^3^	Prism, clear light pink
*Z* = 4	0.20 × 0.20 × 0.07 mm

### catena-Poly[[[(1,4,8,11-tetraazacyclotetradecane-κ4N1,N4,N8,N11)nickel(II)]-µ2-5-carboxybenzene-1,3-dicarboxylato-κ2O1:O3] monohydrate] (II) Data collection

**Table:**

Rigaku Xcalibur Eos diffractometer	4534 independent reflections
Radiation source: fine-focus sealed X-ray tube, Enhance (Mo) X-ray Source	2876 reflections with *I* > 2σ(*I*)
Graphite monochromator	*R*_int_ = 0.042
Detector resolution: 16.1593 pixels mm^-1^	θ_max_ = 26.4°, θ_min_ = 1.9°
ω scans	*h* = −9→11
Absorption correction: multi-scan (CrysAlis PRO; Rigaku OD, 2021)	*k* = −16→18
*T*_min_ = 0.988, *T*_max_ = 1.000	*l* = −19→19
14088 measured reflections	

### catena-Poly[[[(1,4,8,11-tetraazacyclotetradecane-κ4N1,N4,N8,N11)nickel(II)]-µ2-5-carboxybenzene-1,3-dicarboxylato-κ2O1:O3] monohydrate] (II) Refinement

**Table:**

Refinement on *F*^2^	Primary atom site location: dual
Least-squares matrix: full	Hydrogen site location: mixed
*R*[*F*^2^ > 2σ(*F*^2^)] = 0.045	H-atom parameters constrained
*wR*(*F*^2^) = 0.108	*w* = 1/[σ^2^(*F*_o_^2^) + (0.0401*P*)^2^ + 0.0138*P*] where *P* = (*F*_o_^2^ + 2*F*_c_^2^)/3
*S* = 1.03	(Δ/σ)_max_ < 0.001
4534 reflections	Δρ_max_ = 0.32 e Å^−^^3^
287 parameters	Δρ_min_ = −0.43 e Å^−^^3^
0 restraints	

### catena-Poly[[[(1,4,8,11-tetraazacyclotetradecane-κ4N1,N4,N8,N11)nickel(II)]-µ2-5-carboxybenzene-1,3-dicarboxylato-κ2O1:O3] monohydrate] (II) Special details

**Table:**

Geometry. All esds (except the esd in the dihedral angle between two l.s. planes) are estimated using the full covariance matrix. The cell esds are taken into account individually in the estimation of esds in distances, angles and torsion angles; correlations between esds in cell parameters are only used when they are defined by crystal symmetry. An approximate (isotropic) treatment of cell esds is used for estimating esds involving l.s. planes.

### catena-Poly[[[(1,4,8,11-tetraazacyclotetradecane-κ4N1,N4,N8,N11)nickel(II)]-µ2-5-carboxybenzene-1,3-dicarboxylato-κ2O1:O3] monohydrate] (II) Fractional atomic coordinates and isotropic or equivalent isotropic displacement parameters (Å^2^)

**Table:**

	*x*	*y*	*z*	*U*_iso_*/*U*_eq_	
Ni1	0.500000	0.000000	0.500000	0.02584 (15)	
Ni2	0.000000	0.500000	0.500000	0.02872 (16)	
O1	0.4273 (2)	0.05862 (11)	0.60827 (13)	0.0353 (5)	
O3	0.0632 (2)	0.39590 (12)	0.58677 (14)	0.0414 (6)	
O4	−0.1125 (2)	0.36910 (12)	0.66423 (15)	0.0447 (6)	
O2	0.4878 (2)	0.20071 (13)	0.60647 (14)	0.0467 (6)	
O6	−0.1510 (3)	0.05141 (13)	0.75736 (16)	0.0520 (6)	
O5	0.0511 (2)	−0.02739 (14)	0.77650 (18)	0.0554 (7)	
H5	−0.000820	−0.064204	0.794730	0.083*	
N2	0.7014 (3)	−0.01412 (16)	0.57185 (18)	0.0457 (7)	
H2	0.701145	0.020446	0.624409	0.055*	
N4	0.1739 (3)	0.46959 (16)	0.44077 (17)	0.0430 (7)	
H4	0.167340	0.507198	0.389722	0.052*	
N3	0.1428 (3)	0.57669 (15)	0.58082 (17)	0.0471 (7)	
H3	0.171423	0.541563	0.632809	0.057*	
N1	0.4558 (3)	−0.12013 (14)	0.55014 (17)	0.0449 (7)	
H1	0.465282	−0.164283	0.505861	0.054*	
C15	0.0616 (3)	0.11465 (16)	0.71803 (17)	0.0285 (7)	
C14	−0.0002 (3)	0.19775 (16)	0.70186 (17)	0.0298 (7)	
H14	−0.090488	0.209866	0.716643	0.036*	
C16	0.1962 (3)	0.09647 (17)	0.69488 (17)	0.0303 (7)	
H16	0.236652	0.040675	0.704847	0.036*	
C11	0.2702 (3)	0.16131 (16)	0.65700 (17)	0.0277 (7)	
C13	0.0737 (3)	0.26238 (16)	0.66354 (17)	0.0276 (7)	
C12	0.2094 (3)	0.24429 (16)	0.64298 (17)	0.0294 (7)	
H12	0.260129	0.288490	0.619479	0.035*	
C18	0.0012 (3)	0.34979 (17)	0.63708 (19)	0.0315 (7)	
C17	0.4080 (3)	0.13921 (18)	0.62309 (18)	0.0320 (7)	
C19	−0.0245 (4)	0.04389 (19)	0.75310 (19)	0.0342 (7)	
C10	0.2721 (4)	0.5851 (2)	0.5387 (2)	0.0638 (12)	
H10A	0.354096	0.601851	0.580611	0.077*	
H10B	0.257072	0.630564	0.494968	0.077*	
C9	0.3017 (4)	0.4978 (2)	0.4984 (3)	0.0638 (12)	
H9A	0.382015	0.504040	0.466614	0.077*	
H9B	0.327024	0.453820	0.542783	0.077*	
C3	0.8229 (4)	0.0180 (3)	0.5312 (3)	0.0659 (12)	
H3A	0.832111	−0.018523	0.481770	0.079*	
H3B	0.911249	0.012526	0.571549	0.079*	
C4	0.7132 (4)	−0.1070 (2)	0.5974 (2)	0.0665 (12)	
H4A	0.789513	−0.114001	0.645772	0.080*	
H4B	0.737125	−0.142464	0.550269	0.080*	
C8	0.1819 (4)	0.3768 (2)	0.4105 (2)	0.0617 (12)	
H8A	0.194636	0.337322	0.459597	0.074*	
H8B	0.264607	0.370223	0.380824	0.074*	
C2	0.1962 (4)	−0.1130 (3)	0.4971 (3)	0.0791 (14)	
H2A	0.218068	−0.147555	0.448833	0.095*	
H2B	0.105101	−0.134149	0.511227	0.095*	
C1	0.3115 (5)	−0.1306 (2)	0.5727 (3)	0.0694 (13)	
H1A	0.299479	−0.090074	0.618793	0.083*	
H1B	0.300638	−0.190172	0.593296	0.083*	
C6	0.0876 (5)	0.6599 (2)	0.6088 (2)	0.0658 (12)	
H6A	0.066868	0.699100	0.559851	0.079*	
H6B	0.161339	0.687604	0.650001	0.079*	
C7	−0.0465 (5)	0.6485 (2)	0.6492 (2)	0.0715 (13)	
H7A	−0.064849	0.703405	0.677396	0.086*	
H7B	−0.028017	0.603571	0.693318	0.086*	
C5	0.5729 (5)	−0.1376 (2)	0.6218 (2)	0.0699 (13)	
H5A	0.577720	−0.200247	0.634537	0.084*	
H5B	0.553957	−0.106461	0.672815	0.084*	
O1W	−0.1024 (3)	−0.15256 (17)	0.8190 (3)	0.0910 (11)	
H1WA	−0.064587	−0.195140	0.849607	0.137*	
H1WB	−0.191789	−0.155991	0.822448	0.137*	

### catena-Poly[[[(1,4,8,11-tetraazacyclotetradecane-κ4N1,N4,N8,N11)nickel(II)]-µ2-5-carboxybenzene-1,3-dicarboxylato-κ2O1:O3] monohydrate] (II) Atomic displacement parameters (Å^2^)

**Table:**

	*U*^11^	*U*^22^	*U*^33^	*U*^12^	*U*^13^	*U*^23^
Ni1	0.0253 (3)	0.0224 (3)	0.0308 (3)	0.0006 (2)	0.0073 (2)	−0.0004 (2)
Ni2	0.0254 (3)	0.0257 (3)	0.0350 (3)	0.0011 (2)	0.0041 (3)	0.0068 (2)
O1	0.0420 (13)	0.0267 (11)	0.0410 (13)	0.0045 (9)	0.0185 (11)	−0.0043 (9)
O3	0.0428 (14)	0.0343 (11)	0.0494 (14)	0.0083 (10)	0.0142 (11)	0.0191 (10)
O4	0.0421 (14)	0.0413 (12)	0.0554 (15)	0.0157 (10)	0.0229 (12)	0.0162 (10)
O2	0.0449 (15)	0.0371 (12)	0.0647 (16)	−0.0149 (10)	0.0293 (13)	−0.0184 (11)
O6	0.0456 (16)	0.0403 (13)	0.0755 (18)	0.0032 (11)	0.0268 (14)	0.0123 (11)
O5	0.0472 (15)	0.0363 (12)	0.0852 (19)	0.0030 (11)	0.0187 (14)	0.0283 (12)
N2	0.0368 (18)	0.0553 (17)	0.0431 (18)	0.0092 (13)	0.0003 (14)	−0.0133 (13)
N4	0.0342 (17)	0.0477 (15)	0.0477 (18)	0.0095 (13)	0.0082 (14)	0.0201 (13)
N3	0.058 (2)	0.0397 (15)	0.0406 (17)	−0.0138 (13)	−0.0039 (15)	0.0103 (12)
N1	0.069 (2)	0.0270 (13)	0.0444 (18)	−0.0054 (13)	0.0263 (16)	−0.0009 (12)
C15	0.0353 (18)	0.0260 (15)	0.0243 (16)	−0.0008 (13)	0.0046 (14)	0.0023 (11)
C14	0.0329 (18)	0.0288 (15)	0.0294 (17)	0.0026 (12)	0.0105 (14)	0.0021 (12)
C16	0.0395 (19)	0.0246 (15)	0.0271 (16)	0.0040 (13)	0.0056 (14)	−0.0011 (12)
C11	0.0305 (18)	0.0275 (15)	0.0253 (16)	0.0009 (12)	0.0053 (14)	−0.0026 (12)
C13	0.0338 (18)	0.0276 (15)	0.0218 (15)	0.0031 (12)	0.0052 (13)	0.0009 (11)
C12	0.0345 (18)	0.0247 (15)	0.0299 (17)	−0.0015 (12)	0.0077 (14)	0.0024 (12)
C18	0.035 (2)	0.0265 (16)	0.0315 (17)	0.0047 (13)	−0.0001 (15)	0.0008 (12)
C17	0.0337 (19)	0.0302 (17)	0.0315 (17)	0.0001 (14)	0.0025 (15)	−0.0028 (13)
C19	0.041 (2)	0.0280 (17)	0.0348 (18)	0.0018 (14)	0.0103 (16)	0.0022 (12)
C10	0.043 (2)	0.082 (3)	0.060 (3)	−0.031 (2)	−0.015 (2)	0.027 (2)
C9	0.031 (2)	0.085 (3)	0.075 (3)	0.0057 (19)	0.006 (2)	0.032 (2)
C3	0.026 (2)	0.100 (3)	0.073 (3)	−0.003 (2)	0.009 (2)	−0.036 (2)
C4	0.078 (3)	0.069 (3)	0.046 (2)	0.035 (2)	−0.013 (2)	−0.0007 (19)
C8	0.087 (3)	0.047 (2)	0.062 (3)	0.034 (2)	0.046 (3)	0.0191 (18)
C2	0.049 (3)	0.084 (3)	0.113 (4)	−0.037 (2)	0.041 (3)	−0.041 (3)
C1	0.092 (4)	0.047 (2)	0.081 (3)	−0.029 (2)	0.055 (3)	−0.010 (2)
C6	0.107 (4)	0.043 (2)	0.045 (2)	−0.023 (2)	0.002 (2)	0.0031 (17)
C7	0.128 (4)	0.042 (2)	0.047 (2)	0.010 (2)	0.022 (3)	−0.0051 (17)
C5	0.121 (4)	0.045 (2)	0.043 (2)	0.017 (2)	0.012 (3)	0.0146 (17)
O1W	0.0553 (18)	0.0570 (17)	0.170 (3)	0.0166 (14)	0.047 (2)	0.0672 (18)

### catena-Poly[[[(1,4,8,11-tetraazacyclotetradecane-κ4N1,N4,N8,N11)nickel(II)]-µ2-5-carboxybenzene-1,3-dicarboxylato-κ2O1:O3] monohydrate] (II) Geometric parameters (Å, º)

**Table:**

Ni1—O1	2.1242 (19)	C16—H16	0.9300
Ni1—O1^i^	2.1242 (19)	C16—C11	1.388 (4)
Ni1—N2	2.064 (3)	C11—C12	1.384 (3)
Ni1—N2^i^	2.064 (3)	C11—C17	1.509 (4)
Ni1—N1^i^	2.051 (2)	C13—C12	1.388 (4)
Ni1—N1	2.051 (2)	C13—C18	1.518 (4)
Ni2—O3^ii^	2.1129 (18)	C12—H12	0.9300
Ni2—O3	2.1129 (18)	C10—H10A	0.9700
Ni2—N4^ii^	2.050 (3)	C10—H10B	0.9700
Ni2—N4	2.050 (3)	C10—C9	1.510 (5)
Ni2—N3	2.063 (2)	C9—H9A	0.9700
Ni2—N3^ii^	2.063 (2)	C9—H9B	0.9700
O1—C17	1.261 (3)	C3—H3A	0.9700
O3—C18	1.262 (3)	C3—H3B	0.9700
O4—C18	1.243 (3)	C3—C2^i^	1.508 (5)
O2—C17	1.247 (3)	C4—H4A	0.9700
O6—C19	1.205 (3)	C4—H4B	0.9700
O5—H5	0.8200	C4—C5	1.500 (5)
O5—C19	1.314 (4)	C8—H8A	0.9700
N2—H2	0.9800	C8—H8B	0.9700
N2—C3	1.472 (4)	C8—C7^ii^	1.512 (5)
N2—C4	1.463 (4)	C2—H2A	0.9700
N4—H4	0.9800	C2—H2B	0.9700
N4—C9	1.455 (4)	C2—C1	1.508 (5)
N4—C8	1.490 (4)	C1—H1A	0.9700
N3—H3	0.9800	C1—H1B	0.9700
N3—C10	1.473 (4)	C6—H6A	0.9700
N3—C6	1.457 (4)	C6—H6B	0.9700
N1—H1	0.9800	C6—C7	1.503 (5)
N1—C1	1.459 (4)	C7—H7A	0.9700
N1—C5	1.477 (4)	C7—H7B	0.9700
C15—C14	1.393 (4)	C5—H5A	0.9700
C15—C16	1.394 (4)	C5—H5B	0.9700
C15—C19	1.497 (4)	O1W—H1WA	0.8501
C14—H14	0.9300	O1W—H1WB	0.8501
C14—C13	1.389 (4)		
			
O1^i^—Ni1—O1	180.0	C11—C12—H12	119.6
N2—Ni1—O1	88.88 (9)	C13—C12—H12	119.6
N2^i^—Ni1—O1	91.12 (9)	O3—C18—C13	115.2 (3)
N2—Ni1—O1^i^	91.11 (9)	O4—C18—O3	125.9 (3)
N2^i^—Ni1—O1^i^	88.88 (9)	O4—C18—C13	118.9 (3)
N2—Ni1—N2^i^	180.0	O1—C17—C11	115.8 (2)
N1^i^—Ni1—O1^i^	87.34 (8)	O2—C17—O1	125.2 (3)
N1—Ni1—O1	87.34 (8)	O2—C17—C11	118.8 (2)
N1—Ni1—O1^i^	92.66 (8)	O6—C19—O5	123.8 (3)
N1^i^—Ni1—O1	92.65 (8)	O6—C19—C15	122.9 (3)
N1—Ni1—N2	85.31 (11)	O5—C19—C15	113.2 (3)
N1^i^—Ni1—N2^i^	85.31 (11)	N3—C10—H10A	109.8
N1—Ni1—N2^i^	94.69 (11)	N3—C10—H10B	109.8
N1^i^—Ni1—N2	94.69 (11)	N3—C10—C9	109.3 (3)
N1—Ni1—N1^i^	180.00 (16)	H10A—C10—H10B	108.3
O3—Ni2—O3^ii^	180.0	C9—C10—H10A	109.8
N4—Ni2—O3^ii^	92.22 (9)	C9—C10—H10B	109.8
N4—Ni2—O3	87.78 (9)	N4—C9—C10	109.5 (3)
N4^ii^—Ni2—O3	92.22 (9)	N4—C9—H9A	109.8
N4^ii^—Ni2—O3^ii^	87.78 (9)	N4—C9—H9B	109.8
N4^ii^—Ni2—N4	180.0	C10—C9—H9A	109.8
N4^ii^—Ni2—N3	94.62 (11)	C10—C9—H9B	109.8
N4—Ni2—N3	85.38 (11)	H9A—C9—H9B	108.2
N4—Ni2—N3^ii^	94.62 (11)	N2—C3—H3A	109.2
N4^ii^—Ni2—N3^ii^	85.38 (11)	N2—C3—H3B	109.2
N3—Ni2—O3^ii^	94.18 (9)	N2—C3—C2^i^	112.2 (3)
N3^ii^—Ni2—O3^ii^	85.83 (9)	H3A—C3—H3B	107.9
N3—Ni2—O3	85.82 (9)	C2^i^—C3—H3A	109.2
N3^ii^—Ni2—O3	94.18 (9)	C2^i^—C3—H3B	109.2
N3—Ni2—N3^ii^	180.0	N2—C4—H4A	109.8
C17—O1—Ni1	128.73 (18)	N2—C4—H4B	109.8
C18—O3—Ni2	135.10 (19)	N2—C4—C5	109.5 (3)
C19—O5—H5	109.5	H4A—C4—H4B	108.2
Ni1—N2—H2	106.9	C5—C4—H4A	109.8
C3—N2—Ni1	115.6 (2)	C5—C4—H4B	109.8
C3—N2—H2	106.9	N4—C8—H8A	109.4
C4—N2—Ni1	106.0 (2)	N4—C8—H8B	109.4
C4—N2—H2	106.9	N4—C8—C7^ii^	111.1 (3)
C4—N2—C3	114.0 (3)	H8A—C8—H8B	108.0
Ni2—N4—H4	106.6	C7^ii^—C8—H8A	109.4
C9—N4—Ni2	106.7 (2)	C7^ii^—C8—H8B	109.4
C9—N4—H4	106.6	C3^i^—C2—H2A	108.2
C9—N4—C8	113.6 (3)	C3^i^—C2—H2B	108.2
C8—N4—Ni2	116.0 (2)	C3^i^—C2—C1	116.2 (3)
C8—N4—H4	106.6	H2A—C2—H2B	107.4
Ni2—N3—H3	106.4	C1—C2—H2A	108.2
C10—N3—Ni2	105.9 (2)	C1—C2—H2B	108.2
C10—N3—H3	106.4	N1—C1—C2	111.8 (3)
C6—N3—Ni2	116.5 (2)	N1—C1—H1A	109.3
C6—N3—H3	106.4	N1—C1—H1B	109.3
C6—N3—C10	114.6 (3)	C2—C1—H1A	109.3
Ni1—N1—H1	106.6	C2—C1—H1B	109.3
C1—N1—Ni1	116.0 (2)	H1A—C1—H1B	107.9
C1—N1—H1	106.6	N3—C6—H6A	109.0
C1—N1—C5	113.9 (3)	N3—C6—H6B	109.0
C5—N1—Ni1	106.4 (2)	N3—C6—C7	112.8 (3)
C5—N1—H1	106.6	H6A—C6—H6B	107.8
C14—C15—C16	120.0 (2)	C7—C6—H6A	109.0
C14—C15—C19	118.8 (3)	C7—C6—H6B	109.0
C16—C15—C19	121.0 (2)	C8^ii^—C7—H7A	108.2
C15—C14—H14	120.2	C8^ii^—C7—H7B	108.2
C13—C14—C15	119.7 (3)	C6—C7—C8^ii^	116.4 (3)
C13—C14—H14	120.2	C6—C7—H7A	108.2
C15—C16—H16	119.9	C6—C7—H7B	108.2
C11—C16—C15	120.3 (2)	H7A—C7—H7B	107.3
C11—C16—H16	119.9	N1—C5—C4	109.3 (3)
C16—C11—C17	120.4 (2)	N1—C5—H5A	109.8
C12—C11—C16	119.4 (3)	N1—C5—H5B	109.8
C12—C11—C17	120.0 (2)	C4—C5—H5A	109.8
C14—C13—C18	120.1 (3)	C4—C5—H5B	109.8
C12—C13—C14	119.8 (2)	H5A—C5—H5B	108.3
C12—C13—C18	119.8 (2)	H1WA—O1W—H1WB	104.5
C11—C12—C13	120.9 (3)		
			
Ni1—O1—C17—O2	−39.4 (4)	C14—C13—C18—O4	13.4 (4)
Ni1—O1—C17—C11	135.4 (2)	C16—C15—C14—C13	−0.7 (4)
Ni1—N2—C3—C2^i^	54.6 (4)	C16—C15—C19—O6	−164.3 (3)
Ni1—N2—C4—C5	−40.7 (3)	C16—C15—C19—O5	14.1 (4)
Ni1—N1—C1—C2	56.5 (4)	C16—C11—C12—C13	−2.3 (4)
Ni1—N1—C5—C4	−39.1 (3)	C16—C11—C17—O1	20.0 (4)
Ni2—O3—C18—O4	−19.0 (5)	C16—C11—C17—O2	−164.8 (3)
Ni2—O3—C18—C13	159.41 (19)	C12—C11—C17—O1	−153.7 (3)
Ni2—N4—C9—C10	−39.8 (3)	C12—C11—C17—O2	21.5 (4)
Ni2—N4—C8—C7^ii^	55.9 (3)	C12—C13—C18—O3	9.5 (4)
Ni2—N3—C10—C9	−39.6 (3)	C12—C13—C18—O4	−171.9 (3)
Ni2—N3—C6—C7	54.0 (4)	C18—C13—C12—C11	−172.1 (2)
N2—C4—C5—N1	55.0 (4)	C17—C11—C12—C13	171.5 (3)
N3—C10—C9—N4	54.9 (4)	C19—C15—C14—C13	−175.3 (3)
N3—C6—C7—C8^ii^	−70.5 (4)	C19—C15—C16—C11	175.6 (3)
C15—C14—C13—C12	−1.1 (4)	C10—N3—C6—C7	178.4 (3)
C15—C14—C13—C18	173.6 (2)	C9—N4—C8—C7^ii^	−179.8 (3)
C15—C16—C11—C12	0.4 (4)	C3—N2—C4—C5	−169.0 (3)
C15—C16—C11—C17	−173.3 (3)	C3^i^—C2—C1—N1	−71.5 (4)
C14—C15—C16—C11	1.1 (4)	C4—N2—C3—C2^i^	177.9 (3)
C14—C15—C19—O6	10.2 (5)	C8—N4—C9—C10	−169.0 (3)
C14—C15—C19—O5	−171.4 (3)	C1—N1—C5—C4	−168.3 (3)
C14—C13—C12—C11	2.6 (4)	C6—N3—C10—C9	−169.5 (3)
C14—C13—C18—O3	−165.2 (3)	C5—N1—C1—C2	−179.4 (3)

Symmetry codes: (i) −*x*+1, −*y*, −*z*+1; (ii) −*x*, −*y*+1, −*z*+1.

### catena-Poly[[[(1,4,8,11-tetraazacyclotetradecane-κ4N1,N4,N8,N11)nickel(II)]-µ2-5-carboxybenzene-1,3-dicarboxylato-κ2O1:O3] monohydrate] (II) Hydrogen-bond geometry (Å, º)

**Table:**

*D*—H···*A*	*D*—H	H···*A*	*D*···*A*	*D*—H···*A*
O5—H5···O1*W*	0.82	1.72	2.531 (3)	170
N2—H2···O6^iii^	0.98	2.38	3.199 (4)	141
N4—H4···O4^ii^	0.98	2.09	2.959 (3)	147
N1—H1···O2^i^	0.98	1.97	2.872 (3)	153
O1*W*—H1*WA*···O2^iv^	0.85	1.83	2.664 (3)	168
O1*W*—H1*WB*···O4^v^	0.85	1.92	2.747 (3)	165

Symmetry codes: (i) −*x*+1, −*y*, −*z*+1; (ii) −*x*, −*y*+1, −*z*+1; (iii) *x*+1, *y*, *z*; (iv) −*x*+1/2, *y*−1/2, −*z*+3/2; (v) −*x*−1/2, *y*−1/2, −*z*+3/2.

## References

[bb1] Bosnich, B., Tobe, M. L. & Webb, G. A. (1965). *Inorg. Chem.* **4**, 1109–1112.

[bb2] Choi, H. J., Lee, T. S. & Suh, M. P. (1999). *Angew. Chem. Int. Ed.* **38**, 1405–1408. 10.1002/(SICI)1521-3773(19990517)38:10<1405::AID-ANIE1405>3.0.CO;2-H 29711566

[bb3] Choi, H. J., Lee, T. S. & Suh, M. P. (2001). *J. Inclusion Phenom. Macrocyclic Chem.* **41**, 155–162.

[bb4] Choi, H. J. & Suh, M. P. (1998). *J. Am. Chem. Soc.* **120**, 10622–10628.

[bb26] Groom, C. R., Bruno, I. J., Lightfoot, M. P. & Ward, S. C. (2016). *Acta Cryst*. B**72**, 171–179. 10.1107/S2052520616003954 PMC4822653 27048719

[bb5] Lampeka, Ya. D. & Tsymbal, L. V. (2004). *Theor. Exp. Chem.* **40**, 345–371.

[bb6] Lampeka, Ya. D., Tsymbal, L. V., Barna, A. V., Shuĺga, Y. L., Shova, S. & Arion, V. B. (2012). *Dalton Trans.* **41**, 4118–4125. 10.1039/c2dt11980c 22266979

[bb7] Lu, T.-B., Xiang, H., Luck, R. L., Jiang, L., Mao, Z.-W. & Ji, L.-N. (2002). *New J. Chem.* **26**, 969–971.

[bb8] Lu, T.-B., Xiang, H., Luck, R. L., Mao, Z.-W., Wang, D., Chen, C. & Ji, L.-N. (2001). *CrystEngComm*, **3**, 168–169.

[bb9] Macrae, C. F., Sovago, I., Cottrell, S. J., Galek, P. T. A., McCabe, P., Pidcock, E., Platings, M., Shields, G. P., Stevens, J. S., Towler, M. & Wood, P. A. (2020). *J. Appl. Cryst.* **53**, 226–235. 10.1107/S1600576719014092 PMC6998782 32047413

[bb10] Meng, X.-R., Zhong, D.-C., Jiang, L., Li, H.-Y. & Lu, T.-B. (2011). *Cryst. Growth Des.* **11**, 2020–2025.

[bb11] Parsons, S., Jagaln, V. B., Harrison, A., Parkin, A. & Johnstone, R. (2006). *Private Communication*.

[bb12] Rao, C. N. R., Natarajan, S. & Vaidhyanathan, R. (2004). *Angew. Chem. Int. Ed.* **43**, 1466–1496. 10.1002/anie.200300588 15022215

[bb13] Rigaku OD (2021). *CrysAlis PRO*. Rigaku Oxford Diffraction, Yarnton, England.

[bb14] Ryoo, J. J., Shin, J. W., Dho, H.-S. & Min, K. S. (2010). *Inorg. Chem.* **49**, 7232–7234. 10.1021/ic101127z 20690732

[bb15] Sheldrick, G. M. (2015*a*). *Acta Cryst.* A**71**, 3–8.

[bb16] Sheldrick, G. M. (2015*b*). *Acta Cryst.* C**71**, 3–8.

[bb17] Shin, J. W., Kim, D.-W. & Moon, D. (2016). *Polyhedron*, **105**, 62–70.

[bb18] Spek, A. L. (2020). *Acta Cryst.* E**76**, 1–11. 10.1107/S2056989019016244 PMC6944088 31921444

[bb19] Stackhouse, C. A. & Ma, S. (2018). *Polyhedron*, **145**, 154–165.

[bb20] Suh, M. P. & Moon, H. R. (2007). *Advances in Inorganic Chemistry*, Vol. 59, edited by R. van Eldik & K. Bowman-James, pp. 39–79. San Diego: Academic Press.

[bb21] Suh, M. P., Park, H. J., Prasad, T. K. & Lim, D.-W. (2012). *Chem. Rev.* **112**, 782–835. 10.1021/cr200274s 22191516

[bb22] Tadokoro, M., Suda, T., Shouji, T., Ohno, K., Honda, K., Takeuchi, A., Yoshizawa, M., Isoda, K., Kamebuchi, H. & Matsui, H. (2015). *Bull. Chem. Soc. Jpn*, **88**, 1707–1715.

[bb23] Tao, B., Cheng, F., Jiang, X. & Xia, H. (2012). *J. Mol. Struct.* **1028**, 176–180.

[bb24] Tsymbal, L. V., Andriichuk, I. L., Shova, S., Trzybiński, D., Woźniak, K., Arion, V. B. & Lampeka, Ya. D. (2021). *Cryst. Growth Des.* **21**, 2355–2370.

[bb25] Westrip, S. P. (2010). *J. Appl. Cryst.* **43**, 920–925.

